# A Functional Data Method for Causal Dynamic Network Modeling of Task-Related fMRI

**DOI:** 10.3389/fnins.2019.00127

**Published:** 2019-02-27

**Authors:** Xuefei Cao, Björn Sandstede, Xi Luo

**Affiliations:** ^1^Division of Applied Mathematics, Brown University, Providence, RI, United States; ^2^Department of Biostatistics and Data Science, School of Public Health, The University of Texas Health Science Center at Houston, Houston, TX, United States

**Keywords:** brain connectivity, dynamic data analysis, optimization, ordinary differential equations, task-related fMRI

## Abstract

Functional MRI (fMRI) is a popular approach to investigate brain connections and activations when human subjects perform tasks. Because fMRI measures the indirect and convoluted signals of brain activities at a lower temporal resolution, complex differential equation modeling methods (e.g., Dynamic Causal Modeling) are usually employed to infer the neuronal processes and to fit the resulting fMRI signals. However, this modeling strategy is computationally expensive and remains to be mostly a confirmatory or hypothesis-driven approach. One major statistical challenge here is to infer, in a data-driven fashion, the underlying differential equation models from fMRI data. In this paper, we propose a causal dynamic network (CDN) method to estimate brain activations and connections simultaneously. Our method links the observed fMRI data with the latent neuronal states modeled by an ordinary differential equation (ODE) model. Using the basis function expansion approach in functional data analysis, we develop an optimization-based criterion that combines data-fitting errors and ODE fitting errors. We also develop and implement a block coordinate-descent algorithm to compute the ODE parameters efficiently. We illustrate the numerical advantages of our approach using data from realistic simulations and two task-related fMRI experiments. Compared with various effective connectivity methods, our method achieves higher estimation accuracy while improving the computational speed by from tens to thousands of times. Though our method is developed for task-related fMRI, we also demonstrate the potential applicability of our method (with a simple modification) to resting-state fMRI, by analyzing both simulated and real data from medium-sized networks.

## 1. Introduction

In recent years, functional magnetic resonance imaging (fMRI) has become a major tool to investigate dynamic brain networks. Earlier analysis methods focus on inferring brain regions activated by external experimental stimuli, for example using a general linear model approach (Friston et al., 1994). More recently, it becomes important to model the inter-connections between brain regions, sometimes called connectivity analysis of fMRI (see for example a review Smith, 2012).

Generally speaking, there are two types of connectivity modeling: functional connectivity and effective connectivity. Functional connectivity usually models the correlations or dependencies between multiple BOLD (blood-oxygen-level dependent) time series from multiple brain regions. It can be estimated using generic statistical methods, such as correlations, partial correlations (Marrelec et al., 2006), regularized inverse covariance (Banerjee et al., 2006). Effective connectivity, on the other hand, aims to model the neuronal or causal connections between brain regions. Some effective methods can also model the effects of external experimental stimuli. Three major effective modeling approaches are dynamic causal modeling (Friston et al., 2003), structural equation modeling (Mclntosh and Gonzalez-Lima, 1994), and Granger causality analysis (GCA) (Goebel et al., 2003; Roebroeck et al., 2005; Deshpande et al., 2008; Seth, 2010). In particular, Granger causality utilizes a data-driven, “time-lagged” prediction criterion to determine the directionality of connections. Though GCA is usually employed to analyze BOLD signals, recent extensions (David et al., 2008; Wheelock et al., 2014; Grant et al., 2015) enable applications of GCA in the latent neuronal space. A closely related extension of GCA in the latent space is state-space multivariate dynamical systems (MDS) (Ryali et al., 2011, 2016a,b). Dynamic causal modeling (DCM), being the more sophisticated modeling approach, utilizes ordinary differential equation (ODE) models for the neuronal dynamics and hemodynamic response, and it is thus an important tool for understanding the biophysical (or neuronal) connections in the brain. However, it often requires prior knowledge or hypotheses of network connections and stimulus projections to narrow down the model space, and then it resorts to a computationally expensive approach to select the “best" fit model after computing all the candidate models. As the number of brain regions under investigation increases, the number of candidate models which need to be computed increases dramatically. Thus the computational burden of fitting many candidate DCM limits the number of regions (in the order of 10 regions) that can be considered. Therefore, DCM is considered to be a hypothesis-validation approach rather than a data-driven approach (Stephan et al., 2010). Despite this limitation, DCM (Friston et al., 2003) has become a standard approach to investigate the brain mechanisms using fMRI, and has been validated in numerous studies (Penny et al., 2004; Lee et al., 2006; David et al., 2008; Dima et al., 2010; Reyt et al., 2010; Brodersen et al., 2011; Frässle et al., 2015).

Most recently, some progress has been made to refine and extend the standard DCM (Friston et al., 2003) described before. Several methods are developed to improve the biophysical interpretation of the DCM neuronal model, including: (1) two-state DCM (Marreiros et al., 2008) that comprises of excitatory and inhibitory neuronal populations in each brain region/node; (2) nonlinear DCM (Stephan et al., 2008) that accounts for nonlinear interactions of neuronal states between nodes; (3) stochastic DCM (Friston et al., 2011; Li et al., 2011) that allows neuronal fluctuations; (4) canonical microcircuit DCM (Friston et al., 2017) that models four neuronal layers per node. These DCMs bring the neuronal mass modeling closer to the underlying biophysical processes. Extensive validation of these DCMs is less developed, compared with the standard DCM. These extended DCMs also introduce more model parameters which can be a computational burden for model inversion, especially for medium or large networks. These issues are mitigated for analyzing resting-state fMRI. Spectral DCM (Friston et al., 2014; Razi et al., 2015) reduces the computation burden using a resting-state formulation of a linear DCM (without the bilinear term in the standard DCM) in the frequency domain, which essentially avoids computing the time-variant parameters. This approach allows inverting larger DCM networks with dozens of nodes (Razi et al., 2017). For task-related fMRI, a linear DCM in the frequency domain is also inverted efficiently using regression DCM (Frässle et al., 2017), and thus it becomes feasible to compute for hundreds of regions (Frässle et al., 2018).

In the statistical literature, inferring ODE parameters from data has been studied separately for general settings, sometimes called dynamic data analysis (DDA) or functional data analysis of ODE models, mostly for the following observation model

(1) $$\begin{array}{l} y\left(t_{i}\right)=x\left(t_{i}\right)+\text{ϵ}\left(t_{i}\right) \end{array}$$

where the observed data ***y***(*t*_*i*_) equals the noise **ϵ**(*t*_*i*_) plus the underlying latent signal ***x***(*t*_*i*_) generated from a set of ODEs. ***y***(*t*_*i*_) is usually sampled at equally spaced time points *t*_*i*_, *i* = 1, …, *n*. There exist several methods to infer the ODE parameters of interest, including the nonlinear least-square (NLS) method (Bard, 1974; Domselaar and Hemker, 1975; Xue et al., 2010), the two-stage smoothing-based estimation method (Varah, 1982; Brunel, 2008; Gugushvili et al., 2012; Brunel et al., 2014), the principal differential analysis and iterated principal differential analysis (Ramsay, 1996; Poyton et al., 2006; Zhang et al., 2015), the generalized profiling method (Cao and Ramsay, 2009; Qi and Zhao, 2010), the Bayesian approaches (Girolami, 2008; Bhaumik and Ghosal, 2015; Chkrebtii et al., 2016). This observational model used in these methods, however, can only hold for specific neuroimaging techniques, such as electrocorticographic (Zhang et al., 2015). Unfortunately, all these existing DDA methods under this observational model have limited applications in fMRI, because the observed BOLD data depend on the current and past neuronal states, here ***x***(*t*_*i*_). Another complication for applying these DDA methods is that fMRI data are measured on a different time scale than the neuronal states with moderate or small signal-to-noise ratios.

One widely used yet simple model for connecting the neuronal states and BOLD responses is the linear convolution model,

(2) $$\begin{array}{l} y\left(t\right)=\int h\left(s\right)x\left(t-s\right)ds+\epsilon \left(t\right) \end{array}$$

where ***y***(*t*_*i*_) is a noise contaminated convolution of ***x***(*t*_*i*_) and a hemodynamic response function (HRF). Similar convolution formulations were employed before to model the neuronal states (Ryali et al., 2011; Karahanoğlu et al., 2013). Different forms of convolution functions have been proposed in the literature (see for example Friston et al., 1998; Lindquist et al., 2009). Nonetheless, this general HRF convolution approach, adopted by many experimental studies, clearly suggests that the DDA observation model (1) is not valid for fMRI. Therefore, these existing statistical methods for ODE modeling cannot be applied directly.

In this paper, we introduce a new statistical modeling framework for estimating effective connectivity and activations simultaneously from fMRI data. We propose a Causal Dynamic Network (CDN) method using a functional/dynamic data analysis approach, which jointly models the neuronal states modeled by a DCM-type ODE model and the observed BOLD responses. Unlike DCM or its extensions (e.g., Marreiros et al., 2008; Friston et al., 2014; Razi et al., 2017), our optimization-based method and algorithm compute the ODE parameters efficiently in a data-driven fashion, instead of comparing potentially a huge number of candidate ODE models. This approach to consider a more general convolution-based observation model also adds to the vast DDA literature. To the best of our knowledge, this type of data-driven ODE modeling for fMRI has not been considered before, and no existing DDA methods can deal with a convolution observation model. We aim to address both challenges in this paper.

The remainder of the paper is organized as follows. We start with a brief review of DCM and discuss its computational issues. We then introduce our CDN model and estimation method in section 2.2. Section 3.1 illustrates the numerical performance of our model using extensive simulations, including simulations from both CDN and DCM. A real fMRI data analysis is presented in section 3.2.

## 2. Materials and Methods

### 2.1. DCM Revisited

Dynamic causal modeling (DCM) was introduced by Friston et al. (2003). DCM provides a general framework to infer the causal activations and connections in brain networks under experimental stimuli. There are two components in DCM. The first component is a set of ODEs characterizing the variations of neuronal responses based on stimuli as

(3) $$\begin{array}{l} x^{\prime }=f\left(u\left(t\right),x\left(t\right),\theta \right) \end{array}$$

where *x* represents the neuronal states of *d* regions, ***u*** is the input from *J* stimuli, and **θ** is the ODE parameter of interest. It is usually difficult to estimate ***f*** in a general form, and instead, DCM employs a bilinear approximation for this dynamic system as follows

(4) $$\begin{array}{l} \frac{dx\left(t\right)}{dt}=Ax\left(t\right)+\overset{J}{\underset{j=1}{\sum }}u_{j}\left(t\right)B_{j}x\left(t\right)+Cu\left(t\right). \end{array}$$

In this approximation, the entry *A*_*mn*_ in ***A*** = (_*A*_*mn*_)*dd*_ denotes the strength of intrinsic causal connection from the *n*-th region to *m*-th region. ***B***_*j*_ = (_*B*_*mnj*_)*dd*_ denotes the influence of the *j*-th input stimulus *u*_*j*_(*t*) on the directional connection between these two regions. ***C*** = (_*C*_*mj*_)*dJ*_ denotes the effects of *u*_*j*_ on the *m*-th region. The parameters ***A***, ***B***, and ***C*** have important biophysical interpretations. ***A*** models the intrinsic effective connectivity between brain regions, ***B*** models how these connections are influenced by external stimuli, and ***C*** models how brain regions are activated (or suppressed) by stimuli. The second part of DCM involves a set of hemodynamic state equations, motivated by the Balloon–Windkessel model (Buxton et al., 1998). These differential equations describe how neuronal activity induces vasodilatory signals that lead to changes in blood volume and deoxyhemoglobin content. These biophysical changes result, in a non-linear form, the fMRI BOLD measures. The parameters in this set of DCM equations are not usually used or interpreted.

To estimate these parameters, DCM in the first stage employs the expectation maximization (EM) procedure to fit a candidate model, usually with specific zero and nonzero patterns in (***A, B, C***) provided by users. In the second stage, from a list of user-specified models, DCM uses the Bayes factor to select the “best" model for a given dataset or experiment. Because of the expensive Bayesian computations in the first stage, users usually need to restrict the number of candidate models and the complexity of such models (e.g., by restricting the number of brain regions considered in the model) (Penny et al., 2010). Clearly, this DCM approach cannot be made data-driven without incurring a huge computational cost, because the number of all possible models grows at least with the order *O*(2^*d*^^2^), where *d* is the number of brain regions.

### 2.2. Causal Dynamic Networks

#### 2.2.1. Model

We propose the following CDN model

(5) $$\begin{array}{l} \frac{dx\left(t\right)}{dt}=Ax\left(t\right)+\underset{j}{\sum }u_{j}\left(t\right)B_{j}x\left(t\right)+Cu\left(t\right) \end{array}$$

(6) $$\begin{array}{l} y\left(t\right)=\int h\left(s\right)x\left(t-s\right)ds+\epsilon \left(t\right) \end{array}$$

where ***y***(*t*) is a *d* × 1 vector of BOLD signals at time *t* of *d* regions, **ϵ**(*t*) is the error process, *h*(*s*) is a hemodynamic response function. The integration for vectors above should be understood as component-wise. Like DCM, each component of ***y***(*t*) for a brain region depends only on its corresponding neuronal component in ***x***(*t*) for the same region. Here, Equations (5) and (6) are fitted separately to each scan session of each participant, and thus the model parameters should also be interpreted as for one session of one participant. Section 2.2.5 describes the approaches to combine these parameter estimates across sessions and participants into group-level (or population-level) estimates.

Equation (5) is the same as the neuronal state model in the standard DCM (Friston et al., 2003). It uses the same bilinear approximation, and thus the interpretations of the key parameters (***A, B, C***) are comparable to the standard DCM. Our goal is to infer the entries in ***A***, ***B***, and ***C*** and their significance levels in this paper. It is important to note that our estimation approach does not need to prespecify the zero/nonzero patterns in these parameters. In this paper, we will focus on this standard DCM. Several extensions of this DCM exist to account for more complex biophysical processes (for example, Marreiros et al., 2008; Stephan et al., 2008; Friston et al., 2011, 2017; Li et al., 2011). Without parameter reduction, these extensions can lead to increased computational costs. To reduce the computation burden, Frässle et al. (2017, 2018) employ a linear DCM (by setting the bilinear coefficient ***B*** = **0**), and compute only the frequency spectra in order to avoid the estimation of ***x***(*t*) in the time domain.

The neuronal state model (5), originally developed for task-related fMRI, is sometimes referred to as *deterministic* DCM. An extension of this model, called *stochastic* DCM, includes an additive term representing endogenous neuronal fluctuations. Stochastic DCMs have been mostly used for modeling resting-state fMRI (Friston et al., 2011; Li et al., 2011) , under the following simplified form

(7) $$\begin{array}{l} \frac{dx\left(t\right)}{dt}=Ax\left(t\right)+\omega \left(t\right) \end{array}$$

where ω(*t*) represents neuronal fluctuations (and possibly external stimulus input). Without any assumptions for ω(*t*), there exists a trivial and less meaningful solution in which ω(*t*) is simply the temporal derivative of *x*(*t*), shown by setting ***A*** = **0**. Under the smoothness assumption, as pointed out by Friston et al. (2011) and Daunizeau et al. (2012), there exists a mathematical connection between deterministic and stochastic DCMs. One can replace ω(*t*) = ***F**ϕ*(*t*) by a linear combination of a reasonable number of (cosine) basis functions ϕ(*t*), and thus the random fluctuations in stochastic DCM are absorbed by the modeling coefficient ***F*** (similar to ***C***) in a deterministic DCM. In practice, however, this deterministic DCM can yield poor estimation (Daunizeau et al., 2012), and inverting (7) directly is computationally more expensive. Because ***x***(*t*) in resting-state fMRI does not have immediate interpretation, more recent methods usually transform this equation to the spectral domain (Friston et al., 2014; Razi et al., 2017). This transformation essentially avoids estimating ***x***(*t*) (and its associated parameters) to reduce computational costs. Our method introduced later will estimate ***x***(*t*) because it can be interpreted along with the stimulus sequences in task-related fMRI. It is possible to apply our model to resting-state fMRI by setting *B* and *C* to zero. This simplification does not account resting-state noise and requires estimating ***x***(*t*). Despite these concerns, this initial paper will provide preliminary validations of our method in resting-state fMRI, without much modification of our algorithm and incurring excessive computational costs. Comprehensive modeling of resting-state fMRI is beyond the scope of this paper, and will be discussed as future work in section 4.

Equation (6) is the relationship between neuronal states and fMRI BOLD signals where *h* is a hemodynamic response function. A similar formulation is used in Karahanoğlu et al. (2013). This is an approximation for the Balloon ODE model in DCM (Henson and Friston, 2007), and it is used here to avoid the heavy computation in fitting ODEs of less importance. Our method allows any hemodynamic response functions to be used. For simplicity, we employ the widely used canonical hemodynamic response function (HRF) (Friston et al., 1998) in this paper. The canonical HRF is mathematically expressed as γ(16*t*; 6, 1/6)−γ(16*t*; 16, 1/16)/6, where the gamma density function γ(*t*; η, υ) = υ^η^*t*^η−1^exp(−υ*t*)/Γ(η), time *t* is in seconds, and Γ(η) is the gamma function.

Together, Equations (5) and (6) can be interpreted as a latent space model in statistics. Our use of a two-equation model is similar to MDS (Ryali et al., 2011, 2016a,b). However, there is an important difference. The first equation in MDS is for discrete latent signals because it is considered as an extension to GCA, whereas ours is an ODE model for continuous neuronal states. Thus, these two models are fundamentally different in modeling the neuronal states, and thus the model parameters are not equivalent.

FMRI machines usually sample ***y***(*t*) at equally spaced time intervals (usually 1–2 s): *t*_1_, *t*_2_, …, *t*_*i*_, …, *t*_*T*_. When fitting our CDN model to data, we replace Equation (6) in our CDN model with the following observation equation, for *i* = 1, …, *T*,

(8) $$\begin{array}{l} y(t_{i})\;=\;\int h(s)x(t_{i}-s)ds\;+\;\epsilon (t_{i})\\ \;=\;h\;⋆\;x(t_{i})\;+\;\epsilon (t_{i}) \end{array}$$

where ⋆ denotes the convolution operation by the integral above. This observation model is different from the popular model (1) in the statistical literature. This difference warrants a new estimation approach. For example, under the classic observation model (1), a general strategy in many existing methods is to approximate ***x***(*t*) from smoothing ***y***(*t*_*i*_) before estimating the ODE parameters. However, this is clearly not applicable to our observation model or fMRI data. As a separate note, although functional data analysis models (see review Wang et al., 2016), especially functional convolution model (Asencio et al., 2014), may appear in a similar form as Equation (8), their estimation goal is fundamentally different from ours. They assume ***y*** and ***x*** are observed in a regression-type setting, mostly with no intention to estimate the ODE parameters associated with ***x***. Here we aim to estimate latent ***x***(*t*) and more importantly the ODE parameters that generate it. Due to these many differences from existing models, we develop a new estimation approach in the next section.

#### 2.2.2. Estimation

To estimate ***x, A, B, C***, we propose to minimize the following loss

$$\begin{array}{l} l(x,\theta )=\sum_{t_{i}}\|y(t_{i})-h⋆x(t_{i}){\|}^{2}\\ +\lambda \int {\left\|\frac{dx(t)}{dt}-(Ax(t)+\sum_{j}u_{j}(t)B_{j}x(t)+Cu(t))\right\|}^{2}dt \end{array}$$

where θ = [***A B C***], λ > 0 is a tuning parameter, and ||·|| is the ℓ_2_ norm. The first part of the loss function is the data fitting error and the second part is regarded as the fidelity to our dynamic system. The ℓ_2_ norm loss was also employed before in Karahanoğlu et al. (2013), with the same goal to deconvolute the HRF.

Following a functional data analysis technique, we represent *x*(*t*) using truncated basis function expansions. Let ***x***(*t*) = **ΓΦ**(*t*) where **Φ**(*t*) is a *p* × 1 vector with entries denoting the basis function value at *t* and *p* is the number of basis functions. We choose *p* to be reasonably large to avoid modeling bias. We will use the classical cubic spline basis to model the neuronal state responses to smooth stimuli and the piece-wise linear basis for box-car stimuli. Certainly many other choices are possible here. The derivative *d**x***(*t*)/*dt*, integrations, solutions for ODE are approximated by numerical methods (e.g., fourth order Runge-Kutta), following the standard idea of solving ODEs numerically as also suggested in the textbook (Ramsay and Silverman, 2006).

With the basis representation, our loss function becomes

$$\begin{array}{l} l(\text{Γ},\theta )=\sum_{t_{i}}\|y(t_{i})-\text{Γ}[h⋆\text{Φ}(t_{i})]{\|}^{2}\\ \text{+λ }{\int \;\left\|\text{Γ}{\text{Φ}}^{\prime }(t)-(A\text{ΓΦ}(t)+\sum_{j}u_{j}(t)B_{j}\text{ΓΦ}(t)+Cu(t))\right\|}^{2}\;dt. \end{array}$$

The number of parameters in this loss function is *dp*+*d*^2^+*Jd*^2^+*dJ*, and the summands are due to estimating ***x***(*t*), ***A***, ***B***, and ***C*** respectively. Here, we will sacrifice a little mathematical rigor for simplified analysis of the estimation equation. The parameters (***A, B, C***) play similar roles as regression coefficients, and thus they are less identifiable if the predictors in the design matrix [***x***(*t*), *u*_1_***x***(*t*), …, *u*_*J*_***x***(*t*), ***u***(*t*)] exhibit higher colinearity. The colinearity can increase as the network size increases, because the number of predictors included in the model grows quadratically with the predominant order (*J*+1)*d*^2^, far exceeding the fMRI data size growing linearly with *d*. This is especially problematic for estimating ***B***, which could be understood as an interaction term in regression. The predictors associated with ***B*** are products of those ones with ***A*** and ***C***, and thus they can be highly correlated with them. Consider a simple box-car task design as an example, where *u*_*j*_(*t*) = 1 during the *j*th task and zero otherwise. The colinearity is affected by the task duration. The reason is that the ***B*** predictors [*u*_*j*_(*t*)***x***(*t*)] equal to the corresponding ***A*** predictors [***x***(*t*)] within the task window and zero otherwise. Thus, extremely long task duration will make these predictors highly correlated overall because they are identical most of the time. Similarly, *u*_*j*_(*t*)***x***(*t*) can also be highly correlated with *u*_*j*_(*t*) if the duration is extremely short and ***x***(*t*) has small variations within the task window.

Though the loss function *l*(**Γ, θ**) is not jointly convex for (**Γ, θ**), it is easy to see that it is convex in **Γ** given **θ** and vice versa. We thus propose to minimize the loss function using iterative block-wise updates for **Γ** and **θ** respectively, summarized in Algorithm 1. Importantly, the iterative updates are given by the explicit form. This strategy is essentially an application of the alternate convex search algorithm for biconvex optimization (see the review Gorski et al., 2007).

**Table:** Algorithm 1 Estimation for CDN

**Input:** BOLD signal ***y***, stimulus ***u***, basis function **Φ**, tuning parameter λ
Initialize **Γ, θ**
**repeat**
Update **Γ** of *l*(**Γ, θ**) given **θ** by gradient descent with backtracking line search
Solve for the minimizer **θ** of *l*(**Γ, θ**) given **Γ**, see Section 2.2.3
**until** convergence
Return **Γ, θ**

#### 2.2.3. Parameter Updates

We derive the parameter updates in our algorithm. We introduce the following notations in order to illustrate our updating procedure. Φ = (ϕ_1_, …, ϕ_*p*_) is our selected basis for estimating neuronal activity. Let

$$\begin{array}{l} P_{1}[n,m]\;=\;\int_{t_{0}}^{t_{T}}\phi_{n}(t)\phi_{m}^{\prime }(t)dt,\\ \;P_{2}^{j}[n,m]=\int_{t_{0}}^{t_{T}}\phi_{n}(t)u_{j}(t)\phi_{m}^{\prime }(t)dt,\\ P_{3}[j,n]\;=\;\int_{t_{0}}^{t_{T}}u_{j}(t)\phi_{m}^{\prime }(t)dt,\\ \;P_{4}[n,m]=\int_{t_{0}}^{t_{T}}\phi_{n}(t)\phi_{m}(t)dt,\\ P_{5}^{j}[n,m]\;=\int_{t_{0}}^{t_{T}}\phi_{n}(t)u_{j}(t)\phi_{m}(t)dt,\\ \;P_{6}[j,n]=\int_{t_{0}}^{t_{T}}u_{j}(t)\phi_{n}(t)dt,\\ P_{7}^{jk}[n,m]=\int_{t_{0}}^{t_{T}}\phi_{n}(t)u_{j}(t)\phi_{m}(t)u_{k}(t)dt,\\ \;P_{8}^{j}[l,n]=\int_{t_{0}}^{t_{T}}u_{l}(t)\phi_{n}(t)u_{j}(t)dt,\\ P_{9}[n,m]\;=\;\int_{t_{0}}^{t_{T}}u_{n}(t)u_{m}(t)dt. \end{array}$$

Combing these into matrix form, we denote

$$\begin{array}{l} \;\theta =\left[A,B_{1},B_{2},..,B_{J},C\right],\\ \;\Psi_{A}=\;{\left[\Gamma P_{4}\Gamma^{\prime },\Gamma P_{5}^{1}\Gamma^{\prime },...,\Gamma P_{5}^{J}\Gamma^{\prime },\Gamma P_{6}^{\prime }\right]}^{\prime },\\ \Xi_{j}={\left[\Gamma P_{5}^{j}\Gamma^{\prime },\Gamma P_{7}^{1j}\Gamma^{\prime },...,\Gamma P_{7}^{Jj}\Gamma^{\prime },\Gamma {\left(P_{8}^{j}\right)}^{\prime }\right]}^{\prime },\\ \;\Psi_{C}={\left[P_{6}\Gamma^{\prime },P_{8}^{1}\Gamma^{\prime },...,P_{8}^{J}\Gamma^{\prime },P_{9}\right]}^{\prime },\\ \text{ Y}=\left[\Gamma P_{1}^{\prime }\Gamma^{\prime },\Gamma P_{2}^{1}\Gamma^{\prime },...,\Gamma P_{2}^{J}\Gamma^{\prime },\Gamma P_{3}^{\prime }\right]. \end{array}$$

Setting the gradient with respect to θ to zero yields

(9) $$\begin{array}{l} \theta \left(\left[{\text{Ψ}}_{A},{\text{Ξ}}_{1},\ldots ,{\text{Ξ}}_{J},{\text{Ψ}}_{C}\right]\right)=Y \end{array}$$

and thus the update for θ is given by

(10) $$\begin{array}{l} \theta =Y{\left(\left[{\text{Ψ}}_{A},{\text{Ξ}}_{1},\ldots ,{\text{Ξ}}_{J},{\text{Ψ}}_{C}\right]\right)}^{-1} \end{array}$$

One can derive the update for Γ by taking gradient.

#### 2.2.4. Tuning Parameter Selection

To select the tuning parameter λ for our model, we use a cross-validation (CV) procedure as follows. Given two time series ***y*_1_** and ***y*_2_** from the same participant, generated by the same dynamic system (with potentially different inputs), we compute our estimator ${\hat{\theta }}_{\lambda }$ by applying Algorithm 1 to ***y***_1_, using λ on a grid. Based on ${\hat{\theta }}_{\lambda }$, we generate ${\hat{x}}_{2}$ using the ODE with varying initial conditions that yield the cross validation (CV) loss, defined as the smallest $||y_{2}-h⋆{\hat{x}}_{2}\left(t\right)|{|}^{2}$. We then select the λ value that minimizes the CV loss. One can also consider varying *p* in this procedure to minimize the CV loss, but we find that our approach is not sensitive for a reasonably large *p*. For example, we use *p*≥50 in our numerical studies. In principle, this CV procedure can be applied to each participant's data to select a subject-specific tuning parameter λ. In this paper, for simplicity, we select one λ that minimizes the average CV loss across all participants.

#### 2.2.5. Group-Level Estimation

Based on the fitted CDN parameters (***A***_*kq*_, ***B***_*kq*_, ***C***_*kq*_) from session *k* of participant *q*, we in this paper average the parameters across sessions and participant to yield a group-level estimate for (***A, B, C***). We use this simple averaging approach here because our simulated and real datasets contain a small fixed number (sometimes, one) of sessions from a relatively large number of participants. Based on our estimates, various alternative approaches can also be applied to obtain group-level inference. For example, when the within-participant dependence/cross-participant variability is a concern or the number of sessions for each participant varies a lot, one may also apply a mixed effects model to (***A***_*kq*_, ***B***_*kq*_, ***C***_*kq*_) and obtain the population estimate. This mixed effects approach was used before for the group level (or second stage) analysis in task activation studies (Worsley et al., 2002).

#### 2.2.6. Inference and P-Values

Because our model can be computed very efficiently (usually in seconds or a few minutes), we propose to use bootstrap (across subjects) method to assess the significance of each entry in (***A, B, C***). The resulting p-values can be used to pick the final model with only statistically significant entries depending on users' choice of significance levels, without resorting to expensive model selection in DCM. For addressing the multiple comparison issue, popular *p*-value correction methods, such as Bonferroni or false discovery rate (FDR) (Benjamini and Hochberg, 1995) correction, should be used to select an appropriate threshold. In this paper, we will use FDR to correct separately the p-values from each matrix ***A***, ***B***, ***C***, and choose a conservative threshold of 0.01 to further control the overall false discovery rates.

## 3. Results

### 3.1. Simulations

In this section, we evaluate the numerical performance of our CDN method using two types of simulation models: DCM and our statistical model CDN. The former provides a more realistic fMRI data generating model while it is computationally more expensive. The latter is computationally inexpensive and will serve to validate the statistical properties of our algorithm. Whenever possible, we compare with three other methods: GCA, MDS, and DCM.

We use the Python Neuroimaging toolbox (Nitime) for the GCA method, and the generative DCM model from Smith et al. (2011). We fit the DCM model using SPM12. The MDS implementation is included in the paper (Ryali et al., 2016b), and the software code of Patel's tau is obtained through personal communications from Professor Stephen Smith, the first author of Smith et al. (2011). Our CDN method is implemented in Python package cdn-fmri, publicly available from https://pypi.org/project/cdn-fmri/. The grid λ = [0.01, 0.1, 1, 10, 100] is used for our CV tuning parameter selection.

#### 3.1.1. Simulated Data From CDN

We consider three simulation scenarios regarding the ODE parameters in our CDN model:

1. S1: ***B*** = 0 and ***C*** = 0
2. S2: ***B*** = 0
3. S3: ***A*** ≠ 0, ***B*** ≠0 and ***C*** ≠ 0

The first one does not consider either the stimulus activations or the stimulus modified connections, and the second one only excludes the stimulus modified connections. Because GCA models neither of these two effects, we include these two scenarios to provide biased advantages to GCA. The last scenario includes both the stimulus activations and the stimulus modified connections, and is a more realistic model for many task-related fMRI experiments.

For these scenarios, we consider a medium-sized network with 10 nodes with boxcar stimuli and different signal-to-noise (SNR) levels (3, 1, 0.5). We use the piece-wise linear basis. The simulations are repeated 50 times for each setting to represent fMRI data from 50 subjects, assuming for simplicity that they have the same underlying structure of brain connectivity, i.e., θ. We set that repetition time (TR) to be 0.72 s to demonstrate an ideal setting for GCA, because longer TR usually leads to low identification accuracy for directionality (Smith et al., 2011). We also set the ***A***, ***B***, and ***C*** based on the simulation code provided by Smith et al. (2011). To validate the explanation of the colinearity issue in section 2.2.2, we also reduce the task duration by 50% in one examplary S3 scenario.

**Figure 1:**
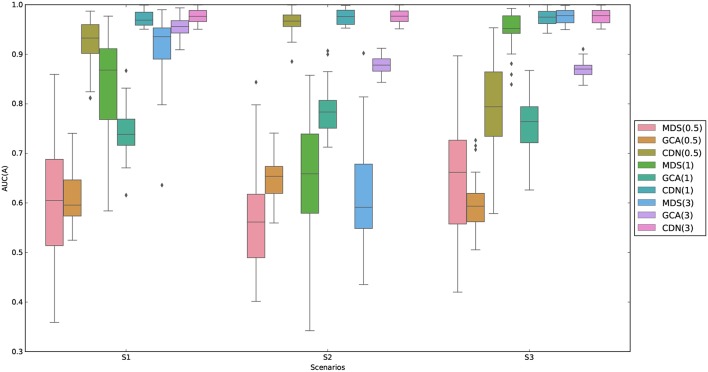
Comparison of AUCs by GCA and CDN for recovering nonzero intrinsic network connections under three simulation scenarios (S1–S3 described in section 3.1.1) and varying signal-to-noise ratios (SNRs). For better visualization, we subtract random uniform jitters (between 0 and 0.05) for those cases when all points equal to 1.

AUC (area under the ROC curve) is used to evaluate the performance of recovering nonzero entries parameters. The AUC is calculated based on comparing the estimates against the true zero/non-zero statuses for all the entries of ***A***. We compare the performance of CDN with GCA and MDS in Figure 1. We are unable to compute the results for DCM here because the computation for this medium-sized network takes more than several days for each replication. It should be noted that the GCA connectivity estimates are based on the BOLD signals while ours are based on the estimated neuronal states. As shown in Figure 1, one can see that CDN performs much better than GCA in identifying the true connections for all SNRs and both stimulus types, partly because CDN models the neuronal states and stimulus activations instead of modeling the BOLD signals only as in GCA. Furthermore, CDN shows robust estimation results across different simulation scenarios. The AUCs of MDS increase as the SNRs increase. It has similar performance as CDN under high SNRs, but has suboptimal performance similar to GCA under low SNRs. Under low SNRs, it also tends to have high variability in AUCs.

To assess the statistical estimation accuracy of CDN, we use AUC and a scaled Frobenius norm for the connectivity-related parameters ***A*** and ***B*** across different scenarios. Because ***B*** is a tensor, we define $||B|{|}_{F}=\sqrt{\underset{m,n,j}{\sum }B_{mnj}^{2}}$. The scaled Frobenious norm is defined as

$$\text{Fro}\left(Ã,A\right)=\frac{||\left(A-Ã\right)|{|}_{F}}{||A|{|}_{F}}$$

where **Ã** is an estimator for the true ***A***. This metric can be interpreted as the relative deviations from the truth.

For the sake of space, we only report the average performance metrics for box-car stimuli with varying SNR (3,1) in Table 1. The results are similar for other settings. This table shows that our CDN algorithm estimates all the model parameters well, and the accuracy improves with increasing SNRs. As explained in section 2.2.2, the accuracy for estimation ***B*** decreases for a shortened task duration.

**Table 1:** Estimation accuracy of CDN under the simulation scenarios described in section 3.1.1.

**Scenario**	**J**	**SNR**	**AUC of A**	**AUC of B**	**AUC of C**	**Fro(A)**	**Fro(B)**	**Fro(C)**
S1	10	0.5	1.00	–	–	0.52	–	–
S1	10	1	1.00	–	–	0.11	–	–
S2	10	0.5	1.00	–	0.98	0.38	–	0.56
S2	10	1	1.00	–	0.98	0.26	–	0.44
S3	1	0.5	0.95	0.71	–	0.55	0.92	0.56
S3	1	1	1.00	0.74	–	0.36	0.88	0.54
S3	1	1 (50% duration)	1.00	0.70	–	0.25	1.07	0.55

*Box-car stimuli are used as a representative example. The accuracy metrics are averaged over 50 replications*.

#### 3.1.2. Simulated Data From DCM

**Figure 2:**
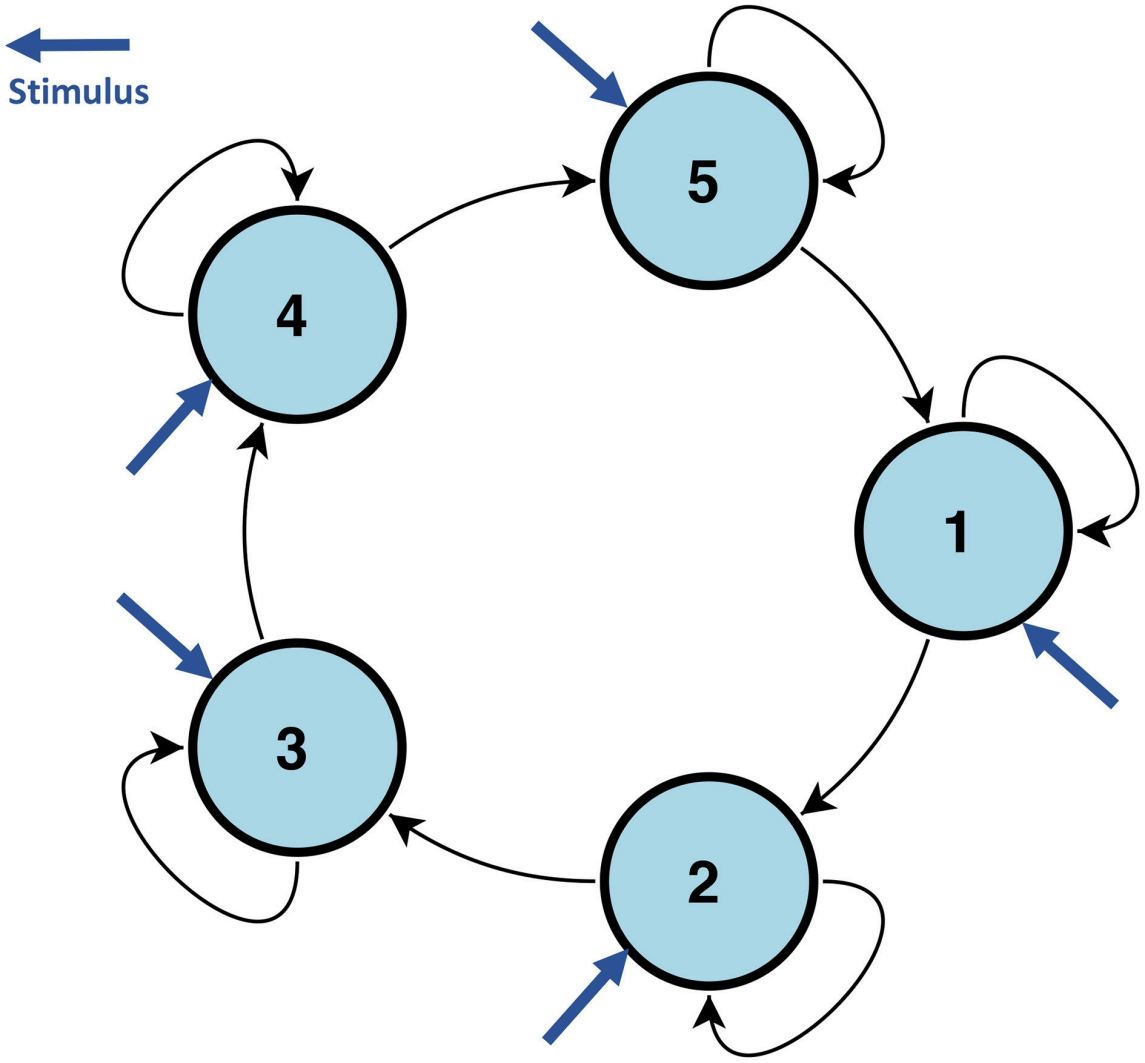
The network structure used to simulate DCM data in section 3.1.2. All nodes are influenced by the same stimulus.

To test the robustness of our proposed model, we also compared the estimation performance using simulated data from DCM. We used the code and a simulation setting from Smith et al. (2011). Due to the heavy computation cost of DCM, we considered a five node brain network with a shared stimulus input. To make this network complex enough to penalize the performance of our method, as shown by the previous simulation study, we let the stimulus input to all nodes, see Figure 2. Due to the high computation cost of DCM, we do not allow the connections to vary with the stimulus, and set ***B*** = 0. We fixed TR=3 s to penalize our method because our CDN model is based on convolutions. To assess the robustness of using a canonical HRF in our model, our simulation setup also includes hemodynamic variability by introducing random perturbations to the ODE model parameters. The same approach is also used by Smith et al. (2011). We simulated 200 subjects with box-car shape stimuli which include five non-overlapped stimuli. We included one session for each stimulus which lasted 70 s.

**Figure 3:**
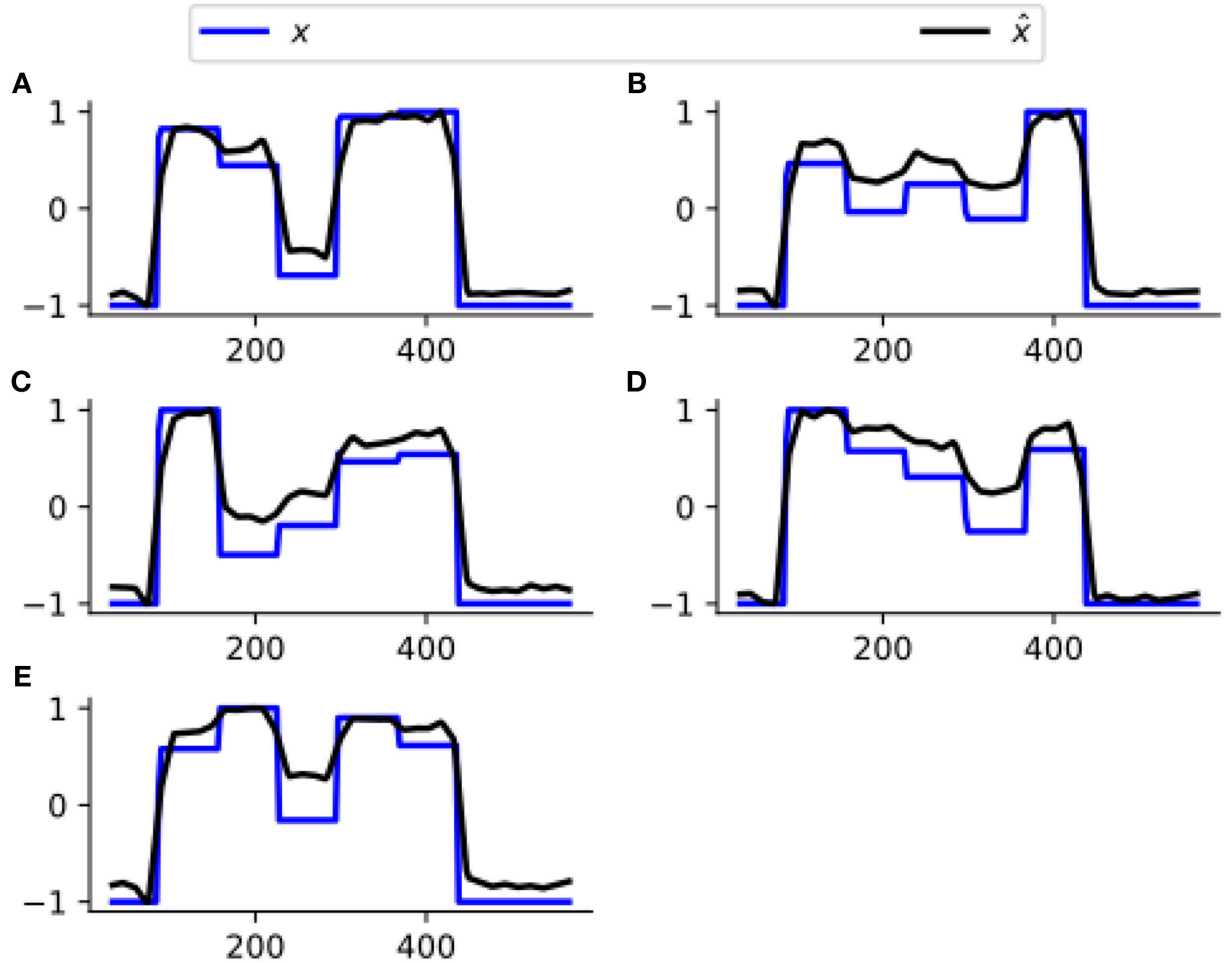
A representative example of the neuronal state time series of five nodes **(A–E)**, simulated by DCM and their estimates by CDN. The DCM network model is plotted in Figure 2. Because neuronal states have arbitrary units, all curves are scaled to have the same minimum and maximum values to illustrate the differences.

Figure 3 assesses the performance of estimating ***x*** from one representative subject's simulated BOLD data. Our CDN estimates capture the main temporal variations in the neuronal states, even though the data are simulated using a relatively large TR. This shows that CDN is robust in recovering the neuronal states, even if the simulation model is different from our CDN model.

Applying DCM usually requires specifying a candidate model of stimulus activation and latent/induced connections. Based on the model, only selected entries in (***A, B, C***) are estimated while all the others are set to zero. To compare the accuracy of estimating latent connections ***A***, we specify ***B*** and ***C*** to have the same pattern as the truth, and let DCM estimate all the entries in (***A*** using a fully connected network model. As before, we use AUC as before to evaluate the estimation accuracy. Table 2 compares the average AUC values for estimating ***A*** by CDN and DCM. CDN yields higher AUC values for both ***A*** using only a fraction of the computation time of DCM.

**Table 2:** Comparison of the AUCs, Frobenius losses, computation times of CDN, DCM and MDS. All computations are conducted in a computer with 8 Intel CPU cores (at 2.6 GHz) and sufficient memory for both algorithms.

**Method**	**AUC (A)**	**Fro loss**	**Computation cost**
**CDN**	**0.77**	**1.29**	**18.95 s**
DCM	0.56	1.42	24 h and 15 min
MDS	**0.77**	1.57	580 s

*The best performance under each comparison criterion is highlighted in bold*.

#### 3.1.3. Simulated Data for Resting-State fMRI

As a simple extension, we use the modification described in section 2.2.1 to fit our model to a simulated resting-state fMRI dataset (simulation scenario 4) from Smith et al. (2011). This dataset contains 50 brain nodes from 50 subjects, and the true network contains 10 subnetworks of size 5. More details on the simulation procedure and parameters are described in (Smith et al., 2011). In the same paper, Patel's τ (Ramsey et al., 2014), an effective connectivity method, was the top performer for recovering the directionality of connections, and thus we also compare with it here.

**Figure 4:**
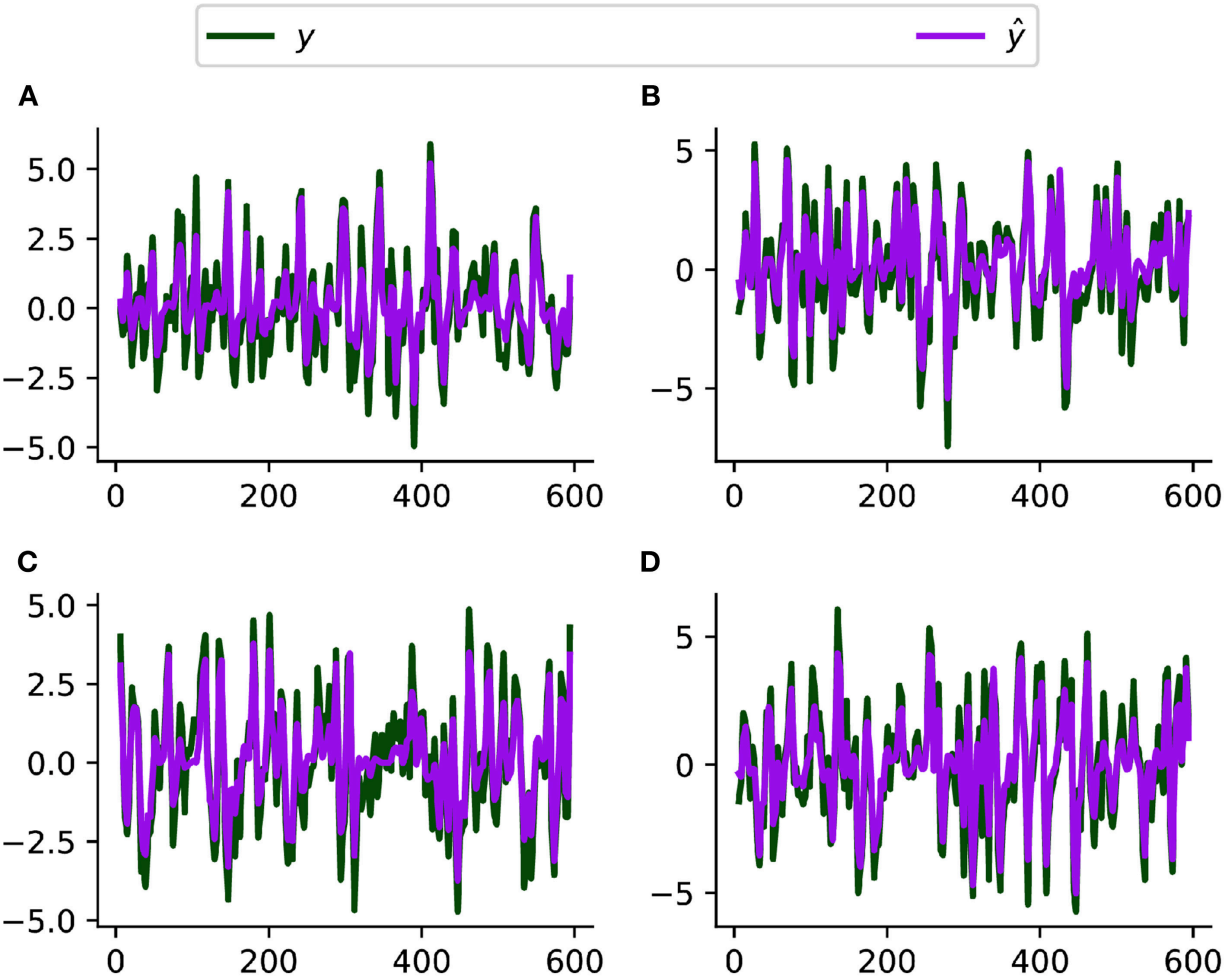
Simulated (*y*) and fitted (ŷ, by CDN) BOLD time series from four representative regions **(A–D)** using a simulated resting-state fMRI dataset from Smith et al. (2011).

Figure 4 compares the simulated and estimated BOLD time series from 4 representative nodes. Our model fits the simulated resting-state fMRI data well overall, though the fitted time series have a slightly smaller variation probably because our method ignores the neuronal fluctuation term. Based on the ROC evaluation metric described before, our method achieves an AUC value of 0.95 for recovering the intrinsic connectivity matrix on this simulated dataset. We are unable to fit the large-scale stochastic DCM method in Razi et al. (2017) to this dataset because of its long algorithmic convergence time. Patel's τ achieves an AUC value of 0.55 on this simulated dataset.

### 3.2. Application

#### 3.2.1. Application to Task FMRI With Block Design

In this section, we apply our method to analyze a block-design fMRI dataset from the Human Connectome Project (HCP). The task is a language processing task developed by Binder et al. (2011). The scan session interleaves 4 blocks of a story task and 4 blocks of a math task. The length of each block varies with an average of approximately 30 s, and the lengths of the story task and math task are roughly matched. The story task asks the participants to classify the topic of the story as revenge or reciprocity for example, after they hear a brief (around 5–9 sentences) story adapted from Aesop's fables, The math task requires the participants to do serial addition and subtraction calculations, and then choose the correct answer. These questions were generated from the same text-to-speech method used in the story task. The participants were then asked to press a button under the right index finger to select the first choice, or a button under the right middle finger to select the second choice. Details about the task design were described in Binder et al. (2011). The HCP task fMRI study, including the imaging protocol, was described in Barch et al. (2013). We analyze the data from 100 examplary subjects, and we follow the suggested HCP preprocessing pipeline (Glasser et al., 2013) to preprocess the data.

We are interested in modeling a language network of regions that were implicated in a previous study (Turken and Dronkers, 2011) using both functional connectivity and diffusion tensor imaging. This network consists of four main regions, including MTG (posterior middle temporal gyrus, MNI: −50, −38, 2), STG (superior temporal gyrus, MNI: −49, 7, −12), STS (posterior superior temporal sulcus, MNI: −45, −62, 21), and IFG (inferior frontal gyrus, pars orbitalis, MNI: −44, 28, −7). After preprocessing the data, we extract the average BOLD time series using 8 mm radius balls centered around these four coordinates. We compute the *p*-values using 10,000 bootstraps. Statistical significance was assessed using a *p*-value threshold of 0.01 after the FDR correction.

**Figure 5:**
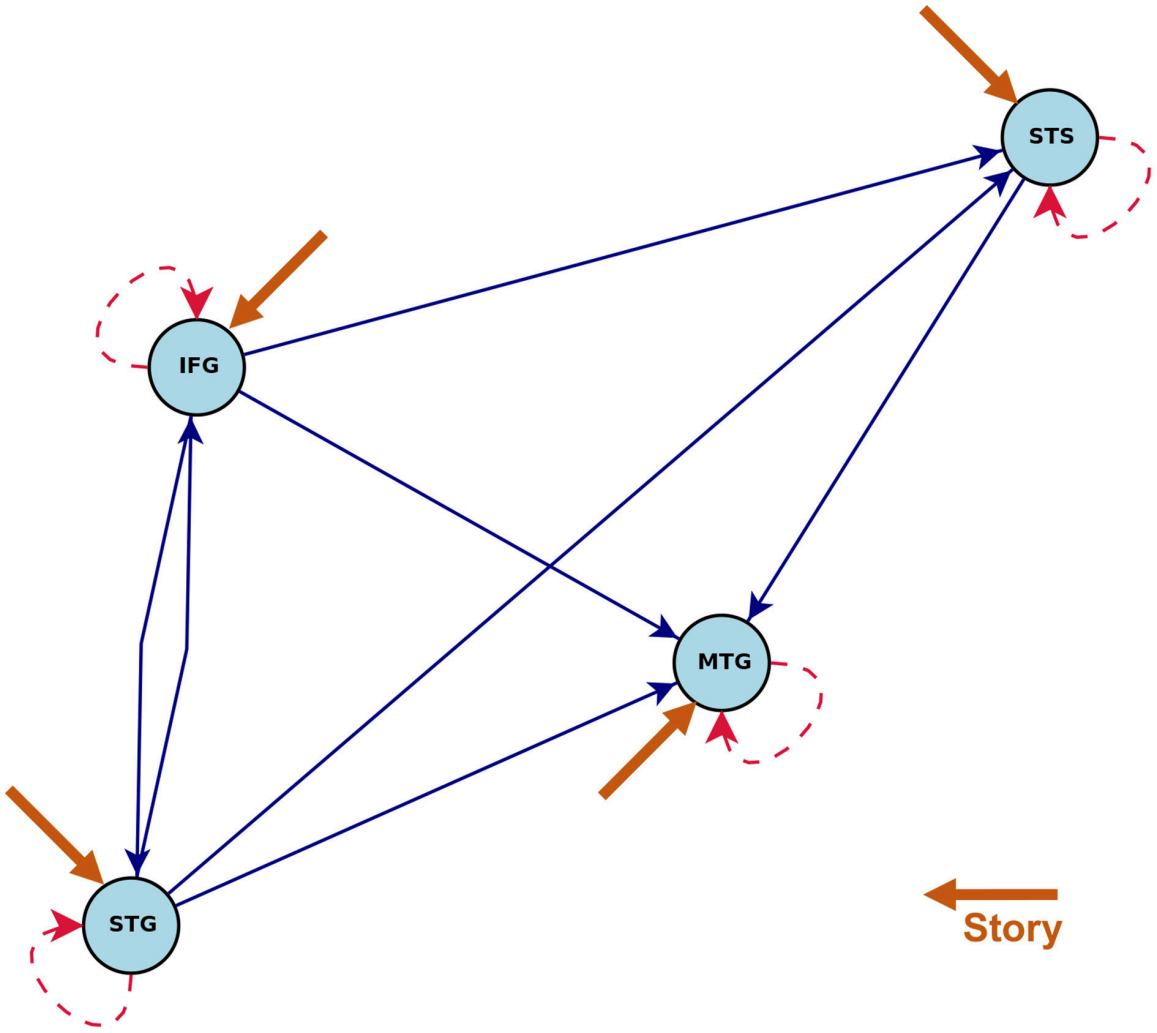
Estimated intrinsic connections in the math-story task experiment. Blue solid lines denote the positive causal connections, and red dashed lines denote negative connections. All connections and positive stimulus projections drawn are statistically significant at level 0.01, FDR corrected.

**Figure 6:**
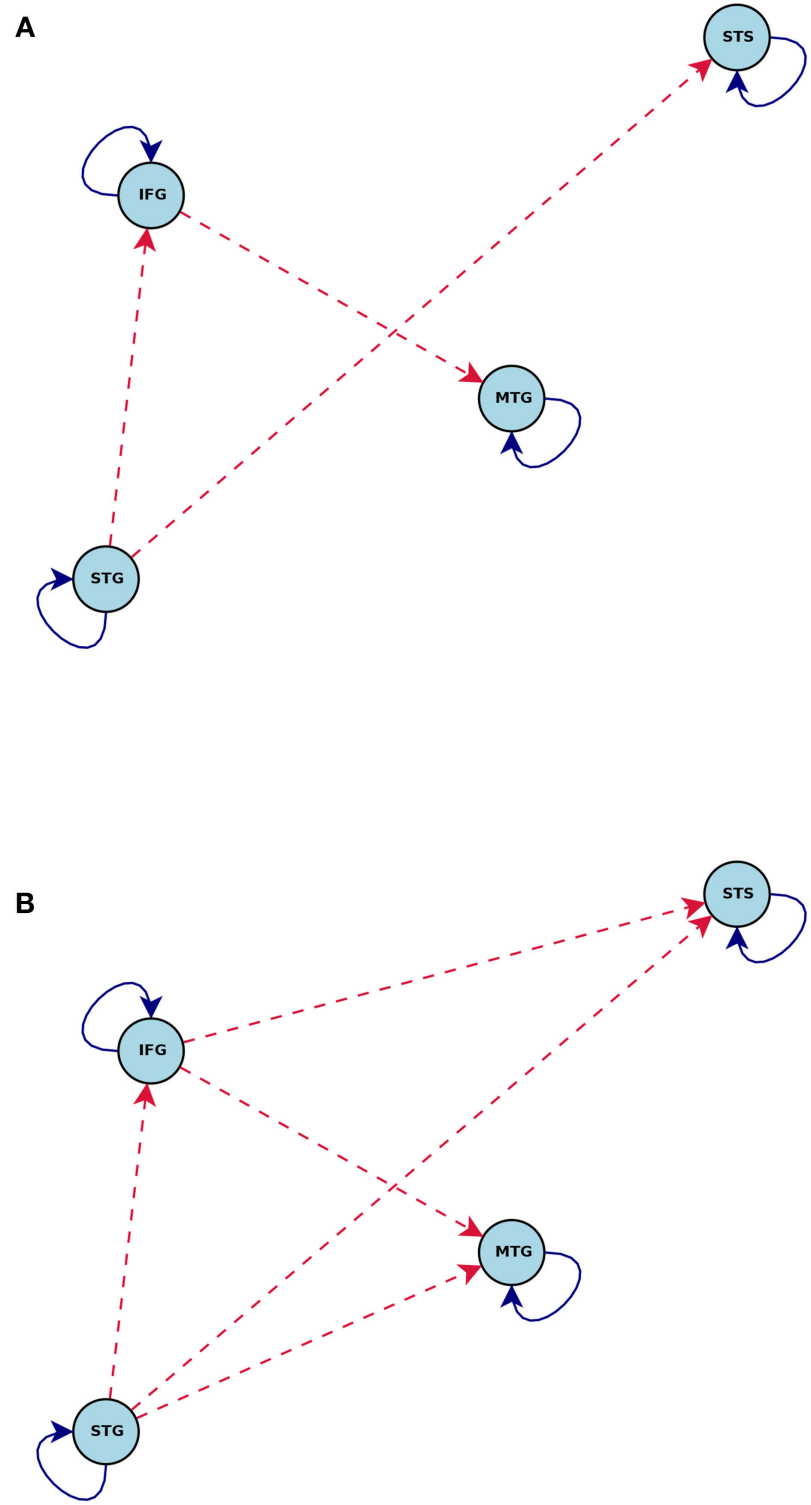
Connections induced by the **(A)** Math and **(B)** Story stimuli estimated by CDN in the math-story task experiment. Blue solid lines denote positive connections and red dashed arrows denote negative connections. All connections drawn are statistically significant at level 0.01, FDR corrected.

Figures 5, 6 show the statistically significant activations and connections estimated by CDN. Our results show that these four ROIs are activated by the story task, but not the math task. This finding is consistent with the previous fMRI activation study (Binder et al., 2011). The non-activation result of the math task fits prior evidence that math calculations do not engage the temporal lobe (Cappelletti et al., 2001; Crutch and Warrington, 2002). Without considering directionality, the recovered intrinsic connections match the functional connectivity findings in Turken and Dronkers (2011). This result showing a well-connected network is consistent with the notion that language comprehension engages a complex network of multiple regions interacting via multiple pathways (Mesulam, 1998;Dronkers et al., 2000).

The directionality recovered by CDN help better elucidate the roles of these ROIs. One key region highlighted by our results is MTG, because it is the converging point of various directional pathways in this network. MTG is regarded as a high-level region contributing to language comprehension. The key role of MTG is supported by various types of prior evidence. For example, MTG was a shared node of six different networks analyzed by a conjunction analysis (Koyama et al., 2010), and it was also found to be also among the most connected node in a resting-state fMRI network analysis of the cerebral cortex (Buckner et al., 2009). A lesion analysis also showed that patients with MTG-lesions perform worse in various language tasks (Dronkers et al., 2004), highlighting its critical role in the language comprehension process. In our CDN results, STG has directional connections pointing to all other three ROIs, suggesting that it may serve as one entry point of this network. Thus, it is likely to be involved in the upstream of the language comprehension process. This implication fits the role of STG in basic morphosyntactic processing (Turken and Dronkers, 2011). Another prior functional imaging evidence supporting this finding is that both speech and complex nonspeech sound activated STG (Wise et al., 1991; Binder et al., 1997).

Comparison of the task-dependent connections (Figure 6) shows that the story task modifies more network connections than the math task. In fact, almost all of the connections are impacted by the story task. This is again consistent with prior evidence that language comprehension engages multiple routes of the language comprehension network (Mesulam, 1998; Dronkers et al., 2000).

#### 3.2.2. Application to Task FMRI With Event-Related Design

We apply our CDN approach to a publicly available event-related fMRI dataset, downloaded from OpenfMRI.org under the access number ds000030. In the experiment, healthy subjects perform the stop-go response inhibition task inside the fMRI scanner. This task consists of two types of trials: go and stop. Specifically, on a go trial, subjects were instructed to press a button quickly when a go stimulus was presented on a computer screen; on a stop trial, subjects were to withhold from pressing when a go stimulus is followed shortly by a stop signal. We preprocess data using a suggested preprocessing pipeline based on FSL, a standard fMRI analysis software. See Poldrack et al. (2016) for a detailed description of the experiment, dataset, and preprocessing steps.

We are interested in studying the regions and their interconnections under either the go or stop stimuli. We select six brain regions implicated in prior publications, which are also validated by the meta-analysis tool from neurosynth.org. These six regions include M1 (primary motor cortex, MNI coordinate: −41, −20, 62), pos-preSMA (posterior presupplementary motor area, MNI: −4, −8, 60), ant-preSMA (anterior presupplementary motor area, MNI: −4, 36, 56), SMA (supplementary motor area, MNI: −3, 6, 50), Thalamus (MNI: −12, −13, 7), and STN (subthalamic nucleus, MNI: 6, −18, −2). The exact brain MNI coordinates for these regions are taken from Aron and Poldrack (2006), Yeo et al. (2011), and Luo et al. (2013). From the data of 100 healthy subjects, we extracted the average BOLD time series using 8 mm radius balls centered around these 6 coordinates. Each time series was standardized to mean zero and unit variance. We selected the tuning parameters using the approach described in section 2.2.4, and computed the p-values using 10,000 bootstraps as described in section 2.2.6. Statistical significance was assessed using a threshold level of 0.01.

**Figure 7:**
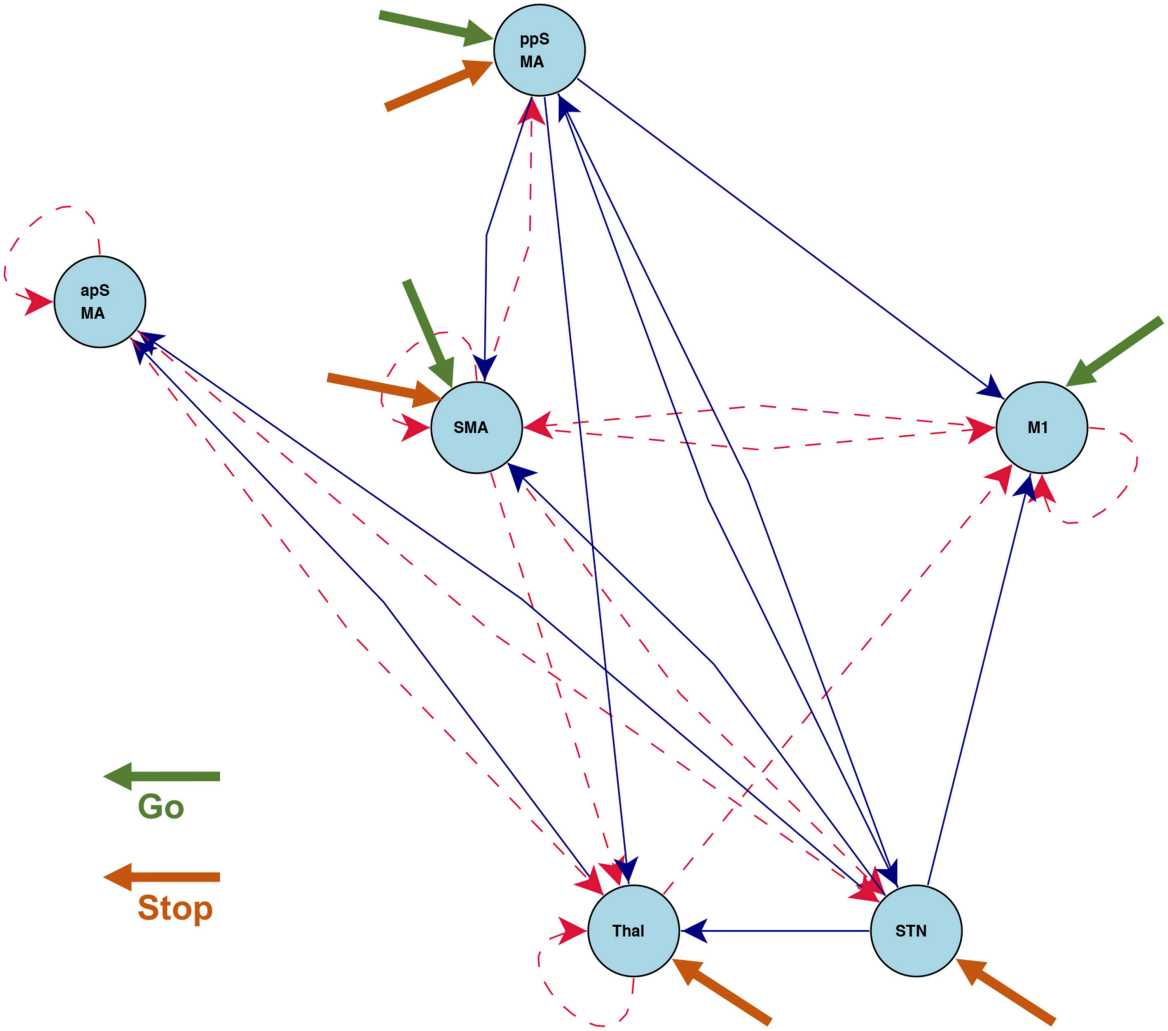
Estimated intrinsic connections in the stop-go task experiment. Blue solid lines denote the positive causal connections, and red dashed lines denote negative connections. All connections and positive stimulus projections drawn are statistically significant at level 0.01, FDR corrected.

Figure 7 shows the statistically significant intrinsic connections and stimulus inputs estimated by CDN. These results identify different connectivity patterns between the anterior and posterior preSMAs. The posterior preSMA is closely connected to Thalamus, STN, and two motor regions (M1 and SMA), while the anterior preSMA is only connected to Thalamus and STN, but not the two motor regions. This finding corroborates the functional connectivity result based on resting-state fMRI in Zhang et al. (2011). Such finding supports the notion that the anterior preSMA is responsible for response inhibition (Haggard, 2008) while the posterior preSMA, along with the SMA, are involved in error detection (Li et al., 2006). Unlike those results from functional connectivity analysis, CDN also infers connectivity directions and stimulus effects, and this leads to a more detailed understanding of these connections and task inputs. For example, M1 receives input from the go stimulus only, while Thalamus and STN receive input from the stop stimulus only. This is well expected from the roles of these regions. M1 is responsible for button pressing under the go stimulus. Thalamus and STN are important regions implicated for response inhibition in several previous fMRI studies, see a review (Aron, 2007). Based the directional connections, CDN recovers a pathway STN → Thalamus → M1. This pathway is consonant with the existing theory and previous fMRI results (Aron, 2007).

**Figure 8:**
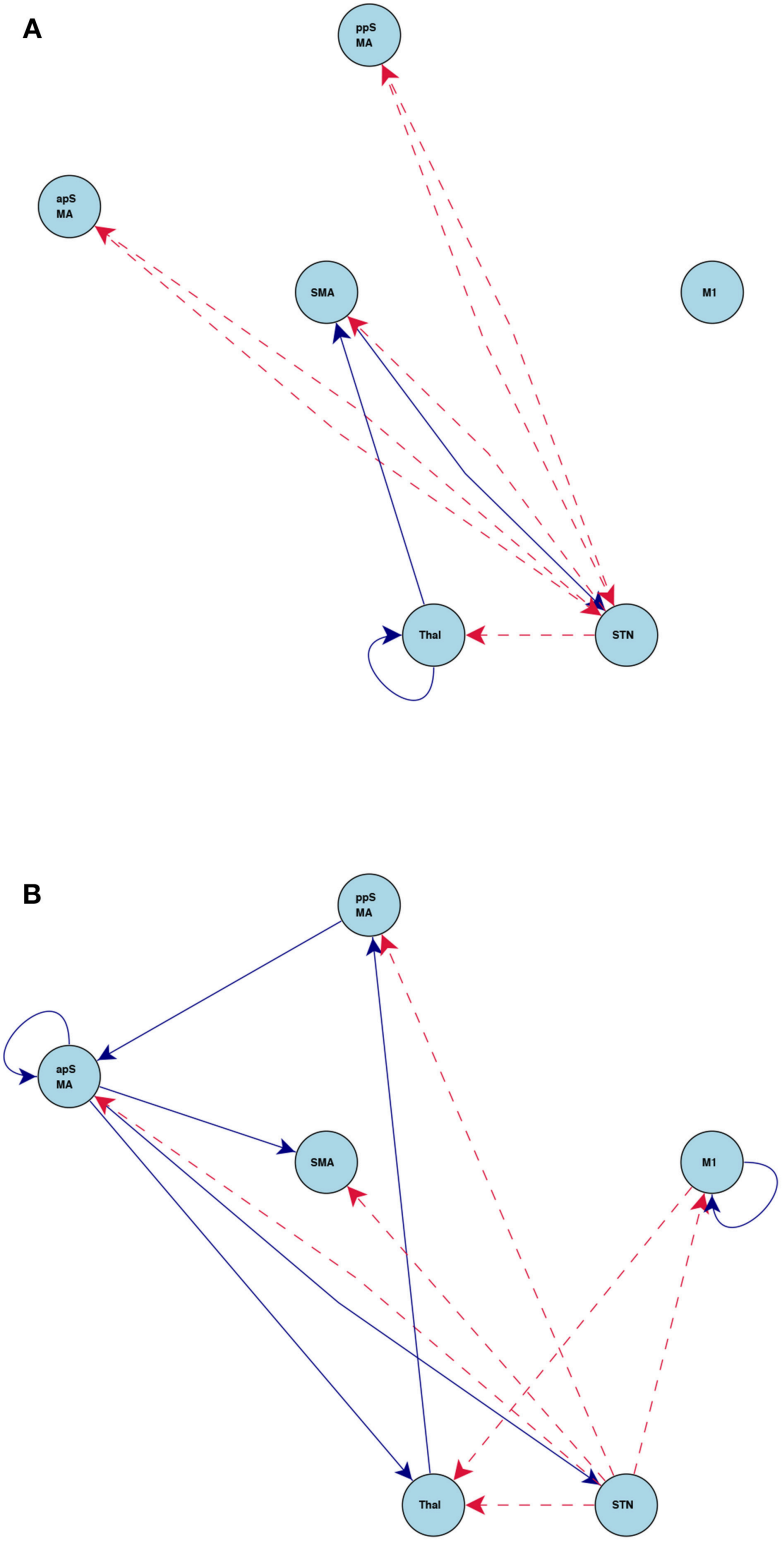
Connections induced by the **(A)** Go and **(B)** Stop stimuli estimated by CDN in the stop-go task experiment. Blue solid lines denote positive connections and red dashed arrows denote negative connections. All connections drawn are statistically significant at level 0.01, FDR corrected.

Figure 8 shows the connections induced by the go and stop stimuli, respectively. Overall, the stop stimulus induces more connectivity strength changes across all the six regions, while go modifies a smaller number of connections. The results on task-induced connections also help better understand the network connection changes under different tasks. For example, CDN identifies a positive connection from the posterior preSMA to the anterior preSMA under stop, while the corresponding intrinsic and go-task dependent connections are not statistically significant. The latter negative finding on effective connectivity replicates the functional connectivity finding in a resting-state fMRI analysis (Zhang et al., 2011). Importantly, the recovered connection from the posterior to anterior preSMA under stop provides an fMRI evidence for the theory that the posterior preSMA detects response conflicts and then projects to the anterior preSMA engaged in response inhibition, see a meta analysis of human neuroimaging studies on the preSMA (Ridderinkhof et al., 2004).

**Figure 9:**
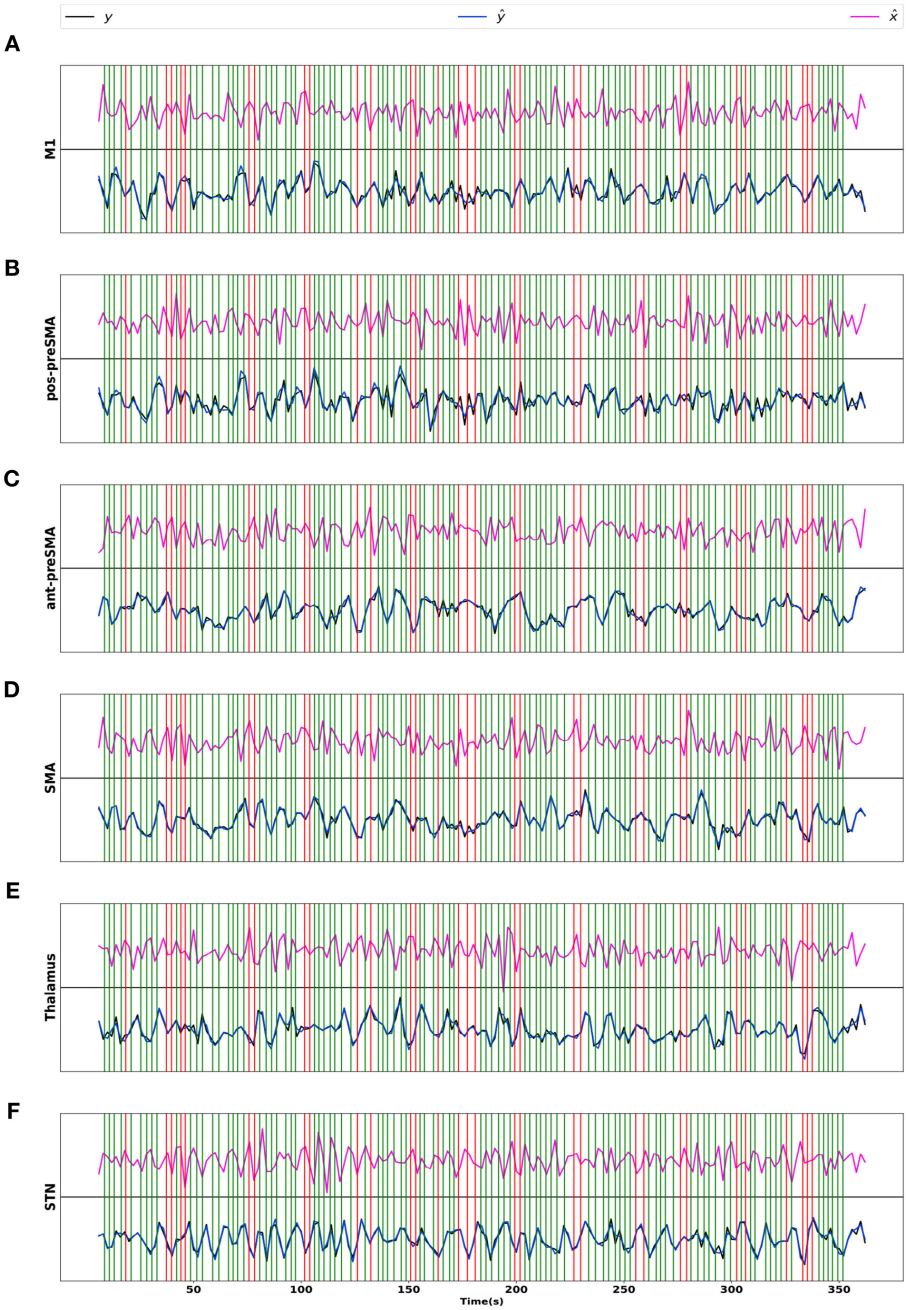
Real BOLD signals (*y*), estimated BOLD signals (ŷ), and neuronal activations ($\hat{x}$) from the STOP/GO experiment, for regions **(A)** M1, **(B)** posterior preSMA, **(C)** anterior preSMA, **(D)** SMA, **(E)** Thalamus, and **(F)** STN. Green/red vertical lines represent the GO and STOP stimuli respectively.

To check the model fit of CDN, we plot the representative BOLD signal and neuronal state time series from all the six regions in Figure 9. The BOLD time series constructed from CDN closely track the variation in the measured BOLD time series. This demonstrates that our CDN model fits the actual data well.

#### 3.2.3. Application to Resting-State fMRI

In this section, we test the applicability of our method for recovering a medium-sized network using resting-state fMRI. The dataset is collected by the Human Connectome Project (Smith et al., 2013), and is preprocessed using a minimal pipeline Glasser et al. (2013). For the sake of manageable computation time, we analyze one scan session from each of 50 selected healthy subjects in this cohort. Each session contains 1,200 scans with a temporal resolution of 0.72 s. We adopt the same 36 ROI coordinates used in a recent large-scale resting-state DCM study Razi et al. (2017). For each ROI, the average BOLD time series from an 8 mm sphere are extracted for analysis. The MNI coordinates and network annotations of these ROIs are listed in Table 3.

**Table 3:** Brain region names and MNI coordinates of the 36 ROIs used in section 3.2.3.

	**Name**	**Coordinates (in mm)**
**DEFAULT MODE NETWORK**		
1	Posterior cingulate/Precuneus	0 −52 7
2	Medial Prefrontal	−1 54 27
3	Left lateral parietal	−46 −66 30
4	Right lateral parietal	49 −63 33
5	Left inferior temporal	−61 −24 −9
6	Right inferior temporal	58 −24 −9
7	Medial dorsal thalamus	0 −12 9
8	Left posterior cerebellum	−25 −81 −33
9	Right posterior cerebellum	25 −81 −33
**DORSAL ATTENTION NETWORK**		
10	Left frontal eye field	−29 −9 54
11	Right frontal eye field	29 −9 54
12	Left posterior IPS	−26 −66 48
13	Right posterior IPS	26 −66 48
14	Left anterior IPS	−44 −39 45
15	Right anterior IPS	41 −39 45
16	Left MT	−50 −66 −6
17	Right MT	53 −63 −6
**CONTROL EXECUTIVE NETWORK**		
18	Dorsal medial PFC	0 24 46
19	Left anterior PFC	−44 45 0
20	Right anterior PFC	44 45 0
21	Left superior parietal	−50 −51 45
22	Right superior parietal	50 −51 45
**SALIENCE NETWORK**		
23	Dorsal anterior cingulate	0 21 36
24	Left anterior PFC	−35 45 30
25	Right anterior PFC	32 45 30
26	Left insula	−41 3 6
27	Right insula	41 3 6
28	Left lateral parietal	−62 −45 30
29	Right lateral parietal	62 −45 30
**SENSORIMOTOR NETWORK**		
30	Left motor cortex	−39 −26 51
31	Right motor cortex	38 −26 48
32	Supplementary motor area	0 −21 48
**VISUAL NETWORK**		
33	Left V1	−7 −83 2
34	Right V1	7 −83 2
**AUDITORY NETWORK**		
35	Left A1	−62 −30 12
36	Right A1	59 −27 15

**Figure 10:**
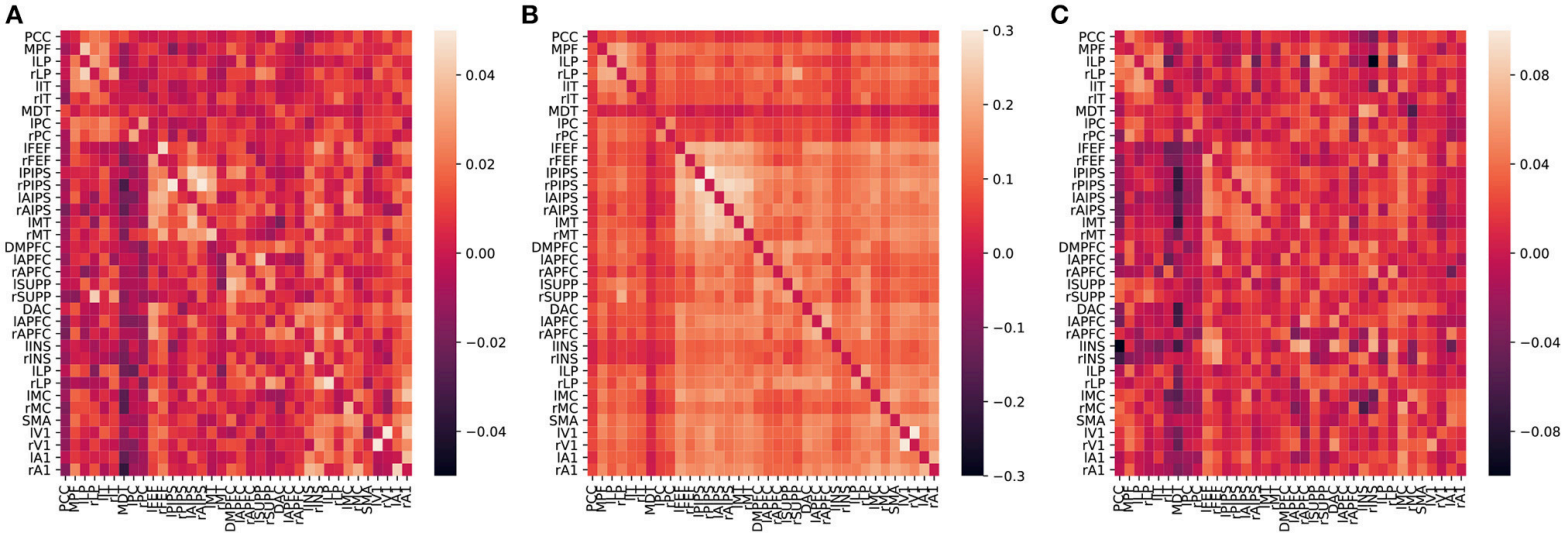
Averaged effective (**A** by CDN, **C** by Razi et al., 2017), functional (**B**, by correlations) connectivity estimates over 50 subjects. The diagonal elements are plotted as zeros in the figure to better illustrate the inter-regional connections.

To assess the validity of our method, Figure 10 compares our CDN estimate with the functional connectivity estimate based on correlations and a large-scale resting-state DCM (lrDCM) (Razi et al., 2017). By visual comparison, our CDN estimate is close to rDCM, especially those connections with large magnitude. Both CDN and lrDCM estimates contain both positive and negative connections, while the correlation estimate shows mostly positive connections. Those strongest connections (in magnitude) are identified by all methods, for example between left and right V1. Our CDN and lrDCM also identify the asymmetry of brain connections while correlations cannot do so.

**Figure 11:**
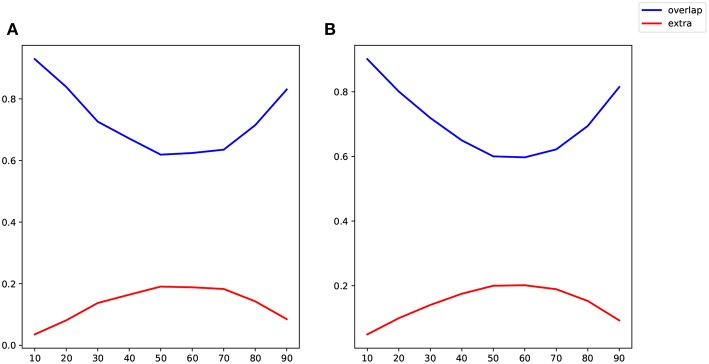
Comparison of identified connections recovered by different methods: **(A)** CDN vs. correlations; **(B)** CDN vs. lrDCM (Razi et al., 2017). Varying thresholds in magnitude are shown on the *x* axis, blue lines show the percentages of connections shared by both methods compared, and red lines show the percentages of nonzero connections in CDN but not in the other method.

Because the values of correlation, CDN, lrDCM estimates are not directly comparable, as they are fitted from different models, we use the following binarization step to convert all the numerical estimates to network connections before the comparison. The rationale is that larger estimated values (in magnitude) in all the models are typically interpreted as stronger connections, and neuroscientists usually resort to these connectivity methods to investigate if connections between certain brain nodes exist or not. Our binarization step converts the largest 100 × *q%* (in magnitude) of off-diagonal entries to 1, and sets the rest to 0, where *q* is a parameter. After converting to binary matrices, we then compute the percentages of network connections found by both methods or one method only. When *q* = 20%, the resulting binary matrices of CDN and lrDCM overlap by 80.09%, while 9.95% of the CDN matrix entries are nonzero while the corresponding lrDCM entries are zero. This shows that CDN and lrDCM yield very similar estimates. It is worth mentioning that our CDN algorithm takes about 10 min in total while lrDCM takes about 19 h with more memory use. The percentages for comparing CDN and correlations are 83.87 and 8.10%, respectively. Figure 11 shows the similar comparison results when *q* varies. Overall, there are large overlaps between the connections recovered by CDN and those by other methods.

## 4. Discussion

We develop a novel causal dynamic method to study brain activations and causal connections simultaneously from fMRI. Driven by the data nature of fMRI, CDN uses a functional data analysis approach to fit ODEs of neuronal states from BOLD time series. Unlike DCM, our model is estimated by an optimization algorithm, and this allows data-driven estimation of the ODE parameters, without DCM's requirement for hypothesizing the connections and stimulus inputs. The high computational efficiency of our algorithm also reduces the computation time.

Based on the task fMRI simulation studies, we show that our CDN approach is robust and accurate for recovering the ODE parameters for modeling dynamic brain networks. Compared with other effective connectivity methods, including GCA, DCM, and MDS, CDN performs the best in terms of parameter estimation and network identification across a wide range of scenarios. In the low SNR settings, CDN has the least variability followed by MDS, suggesting the robustness of our method. In the high SNR settings, both CDN and MDS achieve close to perfect network identification, while CDN yields a smaller parameter estimation loss in a realistic DCM simulation. GCA is relatively sensitive to SNRs, and its performance usually has substantial variability. Regarding the computational speed, CDN requires only a small fraction of the computation times of DCM and MDS. This makes it a very competitive approach for inferring effective connectivity. Patel's tau was a top method for recovering directional connectivity in a previous large-scale simulation study (Smith et al., 2011). However, it only yields a mediocre recovery accuracy on our simulated resting-state dataset. A previous experimental validation (Wang et al., 2017) also found that its accuracy for estimating directionality was not better than chance. Thus, further research is needed to investigate the settings that impact the accuracy of Patel's tau. On the same simulated dataset, CDN achieves much higher network recovery accuracy than Patel's tau. All these comparisons suggest that CDN is an accurate and computationally tractable method for modeling effective connectivity and task activation.

For the analysis of the story/math task fMRI data, our activation and connectivity results are consistent with prior evidence on the language comprehension process and network. Our effective connectivity finding complements the structural and functional connectivity findings (Turken and Dronkers, 2011). Notably, the directional connections in our results help identify the critical regions involved in the process. As we select only a few regions well supported by prior evidence in our analysis, the resulting network is relatively small and may omit other regions and pathways involved. In order to obtain a more comprehensive view of the process, future analysis will need to target a larger number of candidate areas. For the analysis of the stop/go task fMRI, our effective connectivity results corroborate the functional connectivity findings using resting-state fMRI (Zhang et al., 2011), and these findings provide additional support for the existing theory and fMRI results on response inhibition (Ridderinkhof et al., 2004; Aron, 2007). Our CDN results for both activation and connectivity also enhance the understanding of the distinct roles of the anterior and posterior preSMAs, and delineate the pathways involved in response inhibition under different tasks. The network size studied here may also be a limitation because we omit several other regions, including insula, caudate, and the inferior frontal cortex. These regions may mediate or contribute to the response inhibition process (Aron, 2007; Zhang et al., 2011).

CDN in this paper is presented as a purely data-driven method. Other data-driven methods for effective connectivity were proposed in the literature, including latent-space GCA (David et al., 2008; Wheelock et al., 2014; Grant et al., 2015) and state-space multivariate dynamical systems (Ryali et al., 2011, 2016a,b). In order to extend these data-driven methods, it is possible to constrain the network structures and input nodes based on prior knowledge or integrating with other sources of data. For example, one can restrict CDN to fit only those CDN connection parameters between nodes that correspond to significant resting-state functional connectivities (Razi et al., 2017) or anatomical connections (Sotero et al., 2010).

The number of parameters in CDN grows with the numbers of stimuli and nodes. This can be a potential issue to scale our model to large-scale networks with multiple stimuli, especially when the number of parameters exceeds the sample size. This relatively small sample size setting yields a so-called under-determined system, because there might exist multiple sets of parameters that yield the same fit of data. This is further complicated by the colinearity issue introduced by the fMRI task designs. One possible direction is to introduce informative Bayesian priors to help invert such large systems with colinearity. We leave to future research on extending our model for large-scale networks.

The bilinear approximation used in this initial paper is a simplified model for the biophysical mechanisms. It neglects several micro and macro neurophysicological processes (see a review Daunizeau et al., 2011). Therefore, the effective connectivity estimates may be distinct from anatomical connections or synaptic connections, because they fail to model several critical processes, such as gated connections (Stephan et al., 2008), extrinsic and intrinsic connections (Marreiros et al., 2008; Friston et al., 2017), and neuronal fluctuations (Friston et al., 2011; Li et al., 2011). For example, the physiological noise could represent neuronal input from other regions not included in the model or resting-state fluctuations. Without modeling the neuronal noise, the resulting estimated connections between two regions may be indirect ones due to the shared input from other regions. It may also lead to higher estimation errors and over confidence (Daunizeau et al., 2012). Without accounting for these biophysical processes, this bilinear approximation also limits the possibility of combining data from multiple modalities or understanding the nature of the BOLD response (Friston et al., 2017). Though this approximation has been validated before for identifying the existence of effective connections, it may introduce estimation bias on the magnitude of these connections (Friston et al., 2011).

To extend our method to biophysically more realistic models, there remain several challenges for future research. More model parameters associated with these more sophisticated models will increase the computation burden. One should also be aware of the overfitting issue, especially for task-related fMRI where the recording sessions tend to be relatively short with limited repetitions of certain stimuli of interest. There is clearly a trade-off between model complexity and data evidence, in addition to the concern of computation time. Bayesian priors, for example based on anatomical evidence, could be helpful for constraining the model complexity and computation time. However, these priors should be chosen carefully to ensure reliability and robustness (Frässle et al., 2015). We should also note that another direction is to trade biophysical plausibility with large network sizes. For example, Frässle et al. (2017, 2018) replace the bilinear approximation by a linear one in order to estimate large networks from task-related fMRI.

Another limitation of this first paper is its canonical model for HRF. Though our model with the canonical HRF yields relatively robust results for one dataset simulated with HRF variations (Smith et al., 2011), this approach in general may lead to modeling bias. For example, the directionality of connections is determined from the temporal ordering of the neuronal states, as in the ODE model. Such temporal ordering may be reconstructed incorrectly from the observed BOLD signals, if the model does not account for the HRF variations across regions and subjects. Modeling HRF variations remains a challenging topic for various connectivity models as well, including DCM (Handwerker et al., 2012) and functional connectivity (Rangaprakash et al., 2018). In order to mitigate this issue, there are two possible extensions of our approach. One approach is to replace the canonical HRF used in our model by empirically estimated HRF for each region and each subject. A similar approach has been successful in resting-state functional connectivity estimation (Wu et al., 2013). This step serves as data-driven deconvolution of the HRF, in the spirit of those latent-space GCA methods (David et al., 2008; Wheelock et al., 2014; Grant et al., 2015). Because we treat the estimated HRF as given, one can easily adapt our algorithm to fit this extended model. However, to avoid double dipping of the same data, one may need to include a separate scan session to estimate HRF (Aguirre et al., 1998). A natural alternative without extra scanning sessions is to split out one part of the existing time series of each individual, and use that part to estimate the HRF. Either way can be challenging for task fMRI experiment designs, especially when the scanning time may be limited for task fMRI. Further research is also needed to study the optimal allocation of the splits in order to optimally balance the sample sizes available for two estimation steps. Another direction is to treat the HRF as unknown in our model. One will then need to add additional steps in our algorithm to estimate the HRF, for example using flexible functional bases. One may build on the joint estimation framework for detecting brain activation and estimating HRF simultaneously (see Vincent et al., 2010, 2014; Chaari et al., 2013). Future research is needed to extend this framework to incorporate the ODE connectivity model. It is also important to note the possible challenges along this direction. First, though DCM also takes this direction and estimates the HRF empirically, one cannot assume the estimated HRF is accurate (Handwerker et al., 2012). Moreover, these additional parameters may introduce potential estimation issues related to collinearity and identifiability (Vincent et al., 2010). More development, such as introducing regularization (Karahanoğlu et al., 2013) or Bayesian priors (Ryali et al., 2011), may be needed to ensure robust and stable estimation. With these two possible directions and potential issues, we will leave to future research to develop and compare these two possible extensions.

Our method here is mainly developed for task-related fMRI. It shows some promising results on analyzing resting-state fMRI with medium sized networks. However, our method, like others based on the deterministic DCM principal, can be computationally challenging for inverting large-scale networks, partly because our method needs to estimate the hidden neuronal states. For resting-state fMRI, the neuronal states recovered by CDN may serve as input data to other connectivity methods, in order to minimize the confounding effect of HRF variability (Handwerker et al., 2004; Rangaprakash et al., 2018). In the mean time, many large-scale resting-state DCM methods use the spectral domain characterization to avoid the estimation of the neuronal states and associated time-variant parameters (Friston et al., 2014; Razi et al., 2015, 2017; Frässle et al., 2017). One future direction is to develop the spectral domain extension of our method, for analyzing large-scale resting-state fMRI data.

## Data Availability

The stop/go task-related fMRI dataset analyzed for this study can be found on openfmri.org under accession number ds000030 (https://openfmri.org/dataset/ds000030/). The language/math task and resting-state datasets can be found on the Human Connectome Project (https://www.humanconnectome.org).

## Author Contributions

XC and XL designed and carried out the research, drafted the manuscript, analyzed the data, and interpreted the results. XC implemented the new statistical procedure. XL and BS obtained funding, supervised the research, and provided critical revision of the manuscript.

### Conflict of Interest Statement

The authors declare that the research was conducted in the absence of any commercial or financial relationships that could be construed as a potential conflict of interest.
